# Impact of Obesity on Employment and Wages among Young Adults: Observational Study with Panel Data

**DOI:** 10.3390/ijerph16010139

**Published:** 2019-01-07

**Authors:** Hyeain Lee, Rosemary Ahn, Tae Hyun Kim, Euna Han

**Affiliations:** 1 College of Pharmacy, Yonsei University, Incheon 21983, Korea; hnlhailey@gmail.com (H.L.); ahn.rosemary@gmail.com (R.A.); 2 Graduate School of Public Health and Institute of Health Services Research, Yonsei University, Seoul 03722, Korea; 3 College of Pharmacy, Institute of Pharmaceutical Sciences, Yonsei University, Incheon 21983, Korea

**Keywords:** obesity, labor market performance, job qualifications, young adults

## Abstract

This paper assesses the relationship between obesity and the job market by focusing on young adults early on in their careers, while considering the factor of gender and the individuals’ job qualifications. This study extracted data on high school students for four years from the Korean Education and Employment Panel (from 2010 to 2013), a nationally representative dataset comprising of 2000 middle school students and 4000 high school seniors. The individual-level fixed effects were controlled using conditional logistic regression models and an ordinary least squares model. Obese and overweight men were 1.46 times more likely to be placed in professional jobs and had 13.9% higher monthly wages than their normal-weight counterparts. However, obese and overweight women were 0.33 times less likely to have service jobs, earned 9.0% lower monthly wages, and half as likely to have jobs with bonuses than that of their normal-weight counterparts. However, such penalty among women was found only when they had none of the assessed job market qualifications. Given that initial jobs and job conditions have lingering impacts in long-term job performance, the cumulative penalty for overweight or obesity could be more substantial for young adults in particular.

## 1. Introduction

The prevalence of obesity has dramatically increased across the globe and has become a major public health concern [1]. From 1980 to 2013, an increase in obesity prevalence has been evaluated in developed countries from 28.8% to 36.9% for men, from 29.8% to 38.0% for women, from 16.2% to 22.6% for girls, and from 16.9% to 23.8% for boys [2]. This rise has also been observed in newly developed and developing countries where obesity had not been a concern [3]. South Korea is not exceptional in this regard, given that the number of those that are overweight or obese particularly among young adults in the 20s and 30s age group soared from 24.4% in 1998 to 43.8% in 2015 [4,5].

Obesity is a well-recognized risk factor for various illnesses, including cardiovascular diseases and cancer [6]. Being overweight or obese has negative implications in social aspects as well. Several previous studies signify appearance-based discrimination on obese and overweight persons to result in an overall negative effect [7]. For instance, slimness is considered a beauty norm, which accordingly leads obesity to be looked down upon in most modern societies [8,9]. In addition, for the reason that obesity is often controllable at the individual level, it results in the conception of having insufficient self-control and practicing unhealthy behaviors or having poor self-esteem and time management [10,11,12]. Furthermore, one’s overweight or obesity status is visible.

Assuming that employers desire healthy workers with positive social characteristics, all these aspects of overweight or obesity place disadvantages on such individuals in the job market [13]. It has been reported that obese people earn less, are less likely to be hired, or are more likely to remain unemployed [14,15]. Further studies also indicate that obese individuals are less likely to be sorted into jobs that have socially desirable work profiles, such as earning a high salary, positioning in a professional or semi-professional sector, and receiving benefits of high quality in both monetary and non-monetary aspects [16].

The present study builds on previous literature and focuses on the labor market penalty for the obese in their early adulthood life, a period in which individuals are generally new to the labor market, and explored the association between overweight or obesity and job market performance varied by the respondents’ job qualifications. While variables like education can be easily observed, studies find that the measure of the impact of these qualification variables regarding their careers take place after the firms gain access to more information on the productivity of their employees [17]. Likewise, given that young adults tend to be new in their jobs, they do not have the offsetting information that is needed to repel the stigma stemming from obesity [18]. Considering that disadvantage in the labor market early on in their careers may linger later in their career, overweight and obese young adults are more disposed to the negative effects of obesity in the job market [19,20]. Focusing on young adults also gives us leverage to partially mimic experimental settings, since the manifestation of any reverse impacts in job market activities on body mass have yet been realized [6].

There are certain features in a job profile that makes a job aspiring among young adults who are entering the labor market for the first time. The characteristics that comprise of a good job include social prestige, job security such as permanent status and labor union presence, monetary compensation, work contents, and fringe benefits [21]. Although remuneration is the core aspect of a good job, studies indicate that non-pecuniary characteristics also form important attributes that enhance the appeal of a job [21,22,23,24]. Thus, this study also addresses the correlation between overweight or obesity and job market performance by considering both a job’s pecuniary as well as non-pecuniary aspects to comprehensively understand the relationship.

## 2. Materials and Methods

### 2.1. Data

The study obtained data from the Korean Education and Employment Panel (KEEP), a nationally representative dataset comprising of 2000 middle school students and 4000 high school seniors from general high schools and vocational schools. The data gathered via survey comprises of information on the respondents’ education, employment, and household details since 2004, such as parents’ socioeconomic status. This study extracted data on high school students for four years from the Korean Education and Employment Panel (from 2010 to 2013). The response rates of the annual survey during the period from 2010 to 2013 for this study were 76.5%, 75.8%, 76.4%, and 76.4%, respectively. Due to the potential non-randomness of the non-response to the survey, we applied weight on the non-response to adjust the gap between the actual responses and the objective responses. The following exclusion criteria was applied to a total of 9944 person-year observations: missing household information (17 observations), BMI under 10 (one observation) or over 40 (13 observations), and missing income information (40 observations). The final sample included 8340 person-year observations (4714 males and 3626 females), of which 4185 (1959 males and 2226 females) person-years were employed.

### 2.2. Variables

The dependent variable for this study is job performance, which is measured by: (1) employment status, (2) permanent status of one’s job, (3) bonus provision, (4) presence of a labor union, (5) job sector (professional, semi-professional, sales, service, and blue-collar), and (6) monthly wage. Respondents who helped their family at least 18 h per week without payment were considered employed, whereas full-time students and students working as either teaching or research assistants were considered not employed. Temporary employment was defined as either having a job with a labor contract of less than one year or having daily jobs without contracts. Job sector, another measurement of job performance, was categorized based on the South Korean standard classification index (Korean Standard Industrial & Job Classification, 5^th^ edition, 2000.1.7, Statistics Korea) into professional, semi-professional, sales, and service groups, with reference to the blue-collar sector, which comprises of other previously unlisted job classifications like skilled agricultural and fishery workers, installation technicians, and mechanics. Wages were measured on a monthly basis as a linear variable in units of 10,000 Korean Won (KRW). Each dependent variable excluding wage was represented by binary variables.

The key independent variable is obesity status. The study determined the independent variable using the individual’s BMI, which is calculated by dividing the respondents’ self-reported weight in kilograms by the square of the respondents’ self-reported height in meters. Three dummy indicators were generated, representing a clinical classification of BMI: underweight (BMI < 18.5), overweight or obese (BMI ≥ 25), and normal weight (BMI between 18.5 and 25) as reference. Overweight and obesity were combined, considering that the World Health Organization Regional Office for the Western Pacific (WPRO) and Korea Center for Disease Control and Prevention [25] specified BMI ≥ 25 as obese for Asians [26,27]. This study also considered individual qualifications for the job market as independent variables, e.g., having foreign language and other special certificates, job training experiences, or internship experiences. All the qualifications were measured using dummy indicators, with non-experienced as the reference condition.

We controlled the following variables in all estimations as covariates: highest level of educational attainment (high school graduate or below, with reference to college graduate or above), demographic status (gender), health behaviors (cigarette and alcohol use and general health status), personal characteristics (decisiveness and self-restraint), experience of discrimination, and parents’ socioeconomic profiles (employment status and highest education level attained). The ages of the respondents varied by a very small margin because the panel gathered data since the period in which respondents were in their senior year of high school. Furthermore, given that the sample population was in their late twenties, the respondents’ marital status and number of children did not show much variations. Smoking was coded as a binary variable to denote whether the respondents were current smokers. Alcohol usage was measured as drink occasionally and drink frequently, with never drink as the reference. The respondents’ general health statuses were also measured using a dummy variable that represents being healthy and feeling unhealthy, as the reference. Likewise, the respondents’ practice of decisiveness and self-restraint were also measure with dummy indicators.

### 2.3. Estimation

The individual-level fixed effects were controlled using conditional logistic regression models for the dichotomous dependent variables and an ordinary least squares model for a linear measurement of the dependent variable (1):

(1) $$Y_{it}=\beta_{0}+\beta_{1}BMI_{it}+\beta_{2}Qualif_{it}+\beta_{3}X_{it}+\mu_{i}+\varepsilon_{it}$$

where the subscripts *i* and *t* denote individual and year, respectively. *Y* indicates either linear log monthly wages or the following four binary dependent variables: employment, permanent status of the job, bonus provision of the job, and presence of a labor union. The β s indicate estimated parameters. BMI indicates either a linear measurement of BMI or a series of dummy indicators representing underweight and overweight or obese, with normal weight as the reference. Qualif denotes dummy indicators for job qualifications including highest education attainment level, and obtainment of special certificates, job training or internship experiences. *X* is a vector of family information and individual demographic and socioeconomic variables. μ represents individual-level permanent observed and unobserved characteristics, and ε denotes time-varying error term. The unit of analysis was individual-year. For each dependent variable, two separate models were applied, one with a linear measurement of BMI and another wherein BMI was clinically classified into underweight and overweight or obesity (with normal weight as the reference). All estimations were run separately according to sex. Survey weights were applied in all analyses to adjust the unequal selection probability. All statistical analyses were performed using Stata 13.1 (StataCorp, College Station, TX, USA).

## 3. Results

Table 1 shows the distributional characteristics of the final sample by gender. The employment rate was 71.6% for women and 55.11% for men. 

The higher employment rate among women possibly because of the national military service obligation for Korean men, which is approximately three years long and is usually fulfilled in their 20 s. The average monthly wages earned by men and women were 1,781,800 KRW (approximately 1537 USD) and 1,633,380 KRW (approximately 1415 USD), respectively. The majority of the employed had permanent jobs (80% of men and 87% of women). Approximately 61% of men and 58% of women were paid bonuses, but only 21% of men and 16% of women worked in companies with a labor union. There were also gender differences with regard to job qualifications. Women had a larger proportion of internship experiences (42%) than men did (17%), whereas it was the contrary when considering the attainment status of certificates (8% for women and 10% for men). The average BMIs of men and women were 23.31 and 20.20, respectively. Approximately, a quarter of men fell in the overweight or obese category, whereas roughly a quarter of women were in the underweight category. The proportion of women who have reported an experience in discrimination on physical appearance was higher than the proportion for men (8.4% for women versus 5.8% for men). Additionally, an estimate of 60% of men and 50% of women self-assessed that they were decisive or self-restrain in their daily lives.

**Figure 1:**
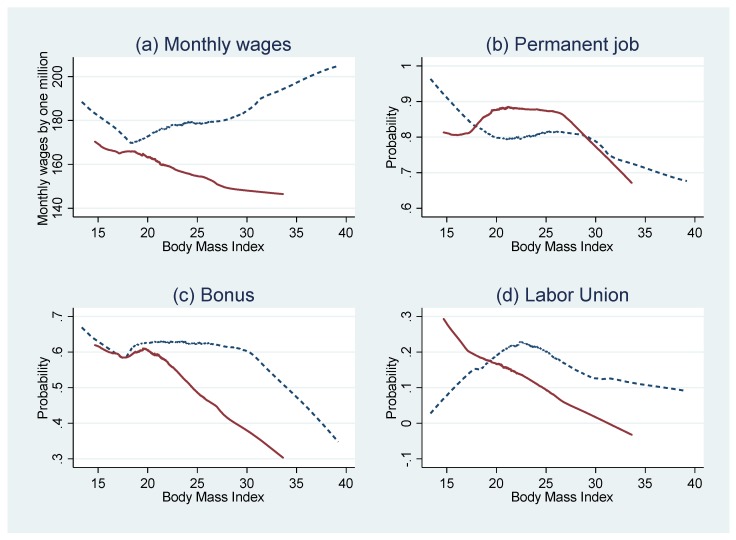
An unadjusted distribution of monthly wages in one million South Korean won and the probabilities of permanent job status, getting a bonus, and having labor union over BMI. Note: The *X*-axis represents body mass index, calculated as weight in kilograms divided by the square of height. The *Y*-axis represents one million Korean won (for monthly wages (**a**)) or probability (for having permanent job (**b**), bonus (**c**), and labor union (**d**)). In both the figures, the solid line is for women and the dotted line is for men.

Figure 1 shows an unadjusted distribution of monthly wages by units of one million KRW (approximately 1000 USD) over BMI. The figure illustrates a negative association between BMI and monthly wages among women, initially showing an ascending pattern up to the average BMI level, following a descending pattern as BMI increases past the average BMI. In the case of men, there was a positive relationship between BMI and monthly wages, showing a descending pattern in the underweight group and a gradual rise in the obese group. Figure 1 also shows the overall penalty in job quality of overweight or obese individuals. As illustrated, the tendency in which an individual has a permanent job, bonus, and assignment in a company that has a labor union dramatically decreases among both men and women with a BMI of 25 or more.

The regression results as shown on Table 2 confirm no statistically significant association of BMI to overweight or obesity and the probability of employment for both genders. Subsequently, the study assessed the association of overweight or obesity with employment in specific job sectors, which consisted of professional, semi-professional, service, sales, and blue-collar sectors. A unit increase in BMI for men was associated with a higher likelihood of having professional jobs by 1.05 times, but a lower likelihood of having service jobs by 0.94 times. An alternative model with a clinical classification of BMI further revealed that there was a positive association between professional jobs and overweight or obese men (by 1.46 times) when compared to that of normal-weight men. On the contrary, men with higher BMI were less likely to belong to service jobs (by 0.94 times) and semi-professional jobs (by 0.50 times), compared to that of normal-weight men.

For women, there were statistically significant associations between the respondents’ body mass status and the likelihood of having a service or sales job. Women with higher BMI were less likely to have service jobs (by 0.83 times), and the magnitude of the negative relationship between the two factors was more apparent for women in the overweight or obese category (by 0.33 times) compared to that of normal-weight women. In contrast, underweight women were nearly half as likely to have blue-collar jobs and were associated with a 13% higher probability of having a blue-collar job per unit increase in BMI (Table 2).

Table 3 shows the association between overweight or obesity and monthly wages. When controlling for time-invariant individual characteristics in a fixed-effect model, overweight or obesity among women was associated with lower monthly wages by 9.0%. Results among men were contrary to that of women, given that a unit increase in BMI was associated with higher monthly wages by 4.6%. An alternative model with a clinical classification of BMI also demonstrated that overweight or obese men had 13.9% higher monthly wages compared to the wages of their normal-weight counterparts (Table 3).

There were statistically significant associations between overweight or obesity and job qualities, particularly among women. Underweight women were 1.57 times more likely to have a permanent job when compared to that of normal-weight women. Similarly, with each unit increase in BMI, women were 0.94 times less likely to have a job with a bonus provision, and overweight or obese women were only half as likely to have jobs with bonuses compared to that of normal-weight women. In addition, overweight or obese women were only 0.39 times likely to have a job in companies that have labor unions than that of their normal-weight counterparts. Such BMI penalties seen in job quality were marginally significant for employed men positioned in companies that have labor unions (Table 4).

Table 5 exhibits the results obtained from subgroup analyses of two groups, where one group comprises of individuals with at the least one job qualification—the possession of foreign language or other special certificates, or experience in job training or internship—and the other group comprises of those without any of the three stated job qualifications. A part of the results revealed that, compared to having none of the three qualifications, having at the least one qualification alleviated penalty for those in the overweight or obesity category. When only considering women with none of the three qualifications, overweight or obesity was estimated to penalize the likelihood of having a permanent job (by 0.35 times compared to their normal-weight counterparts). Similarly, overweight or obese women were 0.33 times likely to have a job with a bonus provision than that of their counterparts. Women in the underweight category were estimated to have rewards for monthly wages in only the subgroup comprising of those without any of the three qualifications.

Based on the analyses on men, overweight or obese men without any qualifications had a 0.81 times less chance of employment compared to that of their counterparts, whereas a statistically significant overweight or obesity penalty was not found for men with at the least one qualification. Overweight or obese men were less likely to have a job in companies that had a labor union compared to that of their counterparts, both in circumstances of having at the least one of the qualifications (by 0.62 times) and in having none of the qualifications (by 0.59 times). Higher monthly wages among overweight or obese men compared to that of their normal-weight counterparts were found only among men with none of the qualifications (Table 5).

## 4. Discussion

Our findings indicate that overweight or obesity had negative associations with labor market performance, particularly for women. The results are similar to those of previous studies showing penalties on overweight or obesity that affect job market performance, particularly among women [15,28,29,30,31,32], and highlight and confirm the existence of such penalties among young adults in their late 20s. Our findings also indicate that a penalty exists not only in the pecuniary aspect but also in other qualitative aspects of a job. For instance, overweight or obese women had job penalties in terms of not only monthly wage level but also job quality features, e.g., permanence status, bonus provision, and labor union presence. However, for men, overweight or obesity was associated with higher monthly wages and a higher likelihood of having professional jobs and enjoying other high quality features such as being part of a labor union.

The present study further explores the extent to which the impact of obesity on job performances is moderated by the level of individual job qualifications, which refers to stipulated abilities used to increase the chance of employment. Job qualifications play a significant role in making hiring decisions because it comprises of a crucial part of the restricted information that firms receive from their applicants. Therefore, firms tend to make judgments by basing off from the average performance for a given job qualification for comparison [33]. Young adults are new to the job market and are more likely to be more prone to the effect of job qualification due to the paucity of evidence of their job capabilities [33].

Education is a representative job qualification and is actively explored for its influence when signaling in the job market [34,35]. The present study expands the observation of job qualifications by adding features that require supplemental personal investments, such as the possession of any certificates, special training for a job, or the completion or presence of internships. This extension is particularly relevant in the context that young adults in South Korea have been substantially investing in extra qualifications other than in formal education, such as internships, foreign language certifications, or short-term coursework abroad—particularly resulting from the recent increase in unemployment rate [36]. The findings of the present study show that the job market penalty for overweight or obesity was mitigated to a certain extent by a boost in job qualifications among young adults. Previous studies also report that job qualifications could moderate poor achievements in the job market for overweight or obese individuals [6,28]. At the same time, other studies find that, regardless of previous experience in job training, obese women face a larger penalty in their job market performance, such as employment outcomes and monthly wages, compared to that of normal-weight women [6,37].

It is also important to acknowledge and address the limitations in the present study. First, since the data was gathered via written questionnaires, height and weight were self-reported. Considering that height is usually over-reported and weight is usually under-reported, the effect of overweight or obesity might be underestimated in this study [38,39]. The existence and extent of these errors cannot be ascertained from the data used in this study. Nevertheless, several studies report that adjusting the gap between the actual and self-reported numbers do not affect the overall estimation results [15,32]. Second, BMI has limited ability to measure actual obesity status particularly in the medical perspective because it does not effectively distinguish fat from fat-free mass such as muscle and bone [40]. Despite this limitation, BMI has several merits including handiness for measurement and high comparability to previous related studies due to its wide use, particularly in the social sciences field [41]. Since there is a limited number of studies that have used fat mass in assessing the impact of obesity on job performance [42], comparisons cannot be accurately constructed between this study and other related studies. BMI is also more affected by visible look than other obesity measurements such as % body fat, and thus, it is useful to assess the relationship between overweight/obesity and job market performance in terms of distaste on appearance in the job market. Third, there is also a potential of reverse causality in our estimations. For example, job performance may reversely influence one’s body mass criteria. Any time-invariant mechanisms for reverse causality were controlled via individual-level, fixed-effect models for both linear and binary dependent variables related to job market performance. However, the presence of any remaining time-varying sources in the error term was not tested.

The study measured job market performance of young adults over a span of four consecutive years. Future studies must explore the evolution of the relationship between overweight or obesity and job market performance throughout their career path, to thoroughly understand the relationship between the two variables and their significance for the general population. Furthermore, comparative studies on the impact of obesity on the job market by different cultural and labor market contexts, both within and across continents, for young adults would also highlight the understanding of mechanisms that underlie in one’s job market performance. Future studies should endeavor to compare differences in the relationship between obesity status and job market performance when obesity is measured based on look versus on actual body fat. Such comparisons will improve our understanding of the mechanism (via obesity-related health versus distaste related to appearance) behind this relationship.

## 5. Conclusions

In summary, this study builds on previous literature by demonstrating the persistence of an association between overweight or obesity and labor market performances in South Korea when holding young adults’ job qualifications independent. Our results imply that the cumulative penalty for overweight or obesity could be more substantial for young adults in particular, given that it is likely of initial jobs and job conditions to have lingering impacts in long-term job performance. The obesity burden on individuals is likely to be spilled over to non-health areas such as the job market, and thus, more proactive public efforts will be needed to control obesity for the younger generations.

## Figures and Tables

**Table 1:** Descriptive statistics.

Variables	Mean (Standard Deviation) (Minimum, Maximum)	
	Men (*N* = 4714)	Women (*N* = 3626)
**Dependent variables**		
Employed	0.5511	0.7168
Monthly wages (10,000 Korean Won) ^a^	178.18 (76.89) (12, 1000)	163.38 (55.96) (15, 500)
Job sector classification ^a^		
Professional/managerial	0.162	0.220
Semi-professional	0.078	0.193
Sales	0.104	0.084
Service	0.230	0.402
Others (reference)	0.423	0.101
Job Quality ^a^		
Permanent position ^b^	0.802	0.870
Having bonus ^b^	0.619	0.587
With labor union ^b^	0.216	0.164
**Independent variable of interest**		
BMI	23.31 (3.15) (13.36, 39.39)	20.20 (2.39) (14.52, 33.62)
BMI Group ^c^		
Underweight	0.024	0.245
Normal weight (reference)	0.728	0.713
Overweight and Obese	0.248	0.042
Qualifications		
Job training ^b^	0.046	0.054
Certificates ^b^	0.101	0.087
Internship ^b^	0.175	0.429
Own education level		
≤High School (reference)	0.616	0.240
College or higher	0.384	0.760
**Other covariates**		
Experience of discrimination based on physical appearance	0.058	0.084
Being Decisive ^b^	0.600	0.492
Being Self-restrained ^b^	0.593	0.495
Self-reported health status: healthy ^b^	0.688	0.490
Currently smoking ^b^	0.444	0.060
Drinking behavior		
Does not drink (reference)	0.196	0.234
Occasional drinker	0.708	0.718
Frequent drinker	0.098	0.051
Father’s socioeconomic status		
High School graduate or lower education ^b^	0.746	0.822
Employed ^b^	0.946	0.937
Mother’s socioeconomic status		
High School graduate or lower education ^b^	0.891	0.927
Employed ^b^	0.635	0.655

^a^ Calculated only for the employed sample (1959 men and 2226 women). ^b^ Dummy indicator. ^c^ BMI groups include underweight (BMI < 25), overweight or obese (BMI ≥ 25), normal weight (18.5 < BMI ≤ 25) as references.

**Table 2:** Results from conditional logistic regression for the association between body mass status and overall employment and job sectors.

Key Independent Variables	Odds Ratio ^b^ (90% Confidence Interval):					
	Employed	Job Sector				
		Professional	Semi-Professional	Service	Sales	Blue-Collar
***MEN***						
Model 1						
BMI	0.988	1.051 **	0.982	0.943 **	1.015	0.991
	(0.97, 1.00)	(1.01, 1.09)	(0.94, 1.03)	(0.90, 0.98)	(0.99, 1.05)	(0.97, 1.02)
Model 2						
Underweight ^a^	1.033	2.036 *	0.312	1.281	0.258 **	1.453
	(0.73, 1.46)	(1.02, 4.07)	(0.06, 1.68)	(0.60, 2.74)	(0.09, 0.70)	(0.77, 2.17)
Overweight & Obese ^a^	0.905	1.469 **	0.501 **	0.983	0.973	0.955
	(0.63, 1.29)	(1.15, 1.87)	(0.42, 0.86)	(0.70, 1.25)	(0.79, 1.20)	(0.80, 1.14)
*N*	*4714*	*1959*	*1959*	*1959*	*1959*	*1959*
***WOMEN***						
Model 1						
BMI	0.996	0.995	0.983	0.833 ***	1.019	1.138 ***
	(0.97, 1.02)	(0.96, 1.03)	(0.94, 1.02)	(0.78, 0.89)	(0.99, 1.05)	(1.08, 1.19)
Model 2						
Underweight ^a^	1.111	1.010	1.100	1.340 *	1.004	0.523 ***
	(0.96, 1.28)	(0.82, 1.24)	(0.89, 1.36)	(1.00, 1.79)	(0.84, 1.20)	(0.37, 0.74)
Overweight & Obese ^a^	0.970	0.220	1.006	0.333 **	0.957	1.450
	(0.70, 1.35)	(0.79, 1.89)	(0.62, 1.63)	(0.14, 0.80)	(0.66, 1.40)	(0.86, 2.44)
*N*	*3626*	*2226*	*2226*	*2226*	*2226*	*2226*

^a^ BMI groups include underweight (BMI < 25), overweight or obese (BMI ≥ 25), normal weight (18.5 < BMI ≤ 25) as references. ^b^ * for *p*-value < 0.1, ** *p*-value < 0.05, *** for *p*-value < 0.01.

**Table 3:** Results from the individual-level fixed-effects model for the association between BMI and log monthly wages.

Key Independent Variable	Regression Coefficient ^b^ (Standard Error) on Log Monthly Wages	
	Women (*N* = 2226)	Men (*N* = 1959)
Model 1		
BMI	0.001	0.046 ***
	(0.007)	(0.009)
Model 2		
Underweight ^a^	−0.003	0.025
	(0.027)	(0.090)
Overweight & Obese ^a^	−0.090 **	0.139 ***
	(0.040)	(0.044)

^a^ BMI groups include underweight (BMI < 25), overweight or obese (BMI ≥ 25), normal weight (18.5 < BMI ≤ 25) as references. ^b^ ** *p*-value < 0.05, *** for *p*-value < 0.01.

**Table 4:** Results from conditional logistic regression models for the association between BMI and job quality.

Key Independent Variable	Odds Ratio ^b^ (90% Confidence Interval):		
	Having A Permanent Job	Getting Bonus in the Job	Having Job in a Company with a Labor Union
***WOMEN***			
Model 1			
BMI	1.043	0.949 ***	0.905 ***
	(0.99, 1.09)	(0.92, 0.98)	(0.86, 0.95)
Model 2			
Underweight ^a^	1.579 ***	1.010	0.748
	(1.25, 2.00)	(0.85, 1.20)	(0.60, 0.93)
Overweight & Obese ^a^	1.125	0.501 ***	0.390 **
	(0.66, 1.92)	(0.34, 0.74)	(0.20, 0.74)
***N***	*2226*	*2226*	*2226*
***MEN***			
Model 1			
BMI	0.998	0.982	0.965 *
	(0.96, 1.03)	(0.96, 1.01)	(0.94, 0.99)
Model 2			
Underweight ^a^	0.502	1.032	2.156
	(0.23, 1.08)	(0.77, 2.19)	(0.97, 4.78)
Overweight & Obese ^†a^	0.615	1.305	1.360
	(0.28, 1.34)	(0.76, 2.23)	(0.60, 3.07)
***N***	*1959*	*1959*	*1959*

^a^ BMI groups include underweight (BMI < 25), overweight or obese (BMI ≥ 25), normal weight (18.5 < BMI ≤ 25) as references. ^b^ * for *p*-value < 0.1, ** *p*-value < 0.05, *** for *p*-value < 0.01.

**Table 5:** Subgroup analyses by job qualification status for the association between BMI and job quality using conditional logistic regression or fixed-effects ordinary least squares.

Key Independent Variables	Odds Ratio ^b^ (90% Confidence Interval):			Regression Coefficient ^b^ (Standard Error):	
	Employed	Permanent	Bonus	Labor Union	Wage
***MEN WITH AT THE LEAST ONE QUALIFICATION*^c^**					
BMI group					
Underweight ^†^	1.196	1.573	0.486	0.445	0.061
	(0.65, 2.21)	(0.40, 6.25)	(0.18, 1.26)	(0.12, 1.62)	(0.157)
Overweight & Obese ^†^	0.988	1.110	1.144	0.624 **	0.087
	(0.81, 1.21)	(0.77, 1.60)	(0.87, 1.50)	(0.46, 0.85)	(0.067)
*N*	*1912*	*920*	*920*	*920*	*920*
***MEN WITHOUT ANY QUALIFICATION ^c^***					
BMI group					
Underweight ^†^	0.896	2.003	0.854	0.479	−0.030
	(0.59, 1.37)	(0.80, 5.03)	(0.46, 1.60)	(0.17, 1.34)	(0.127)
Overweight & Obese ^†^	0.813 **	1.316	0.899	0.597 **	0.196 ***
	(0.69, 0.95)	(0.96, 1.81)	(0.70, 1.16)	(0.42, 0.84)	(0.065)
*N*	*2802*	*1039*	*1039*	*1039*	*1039*
***WOMEN WITH AT THE LEAST ONE QUALIFICATION*^c^**					
BMI group					
Underweight ^†^	0.772 **	1.573	1.082	1.313	−0.038
	(0.62, 0.96)	(0.40, 6.25)	(0.85, 1.37)	(0.98, 1.77)	(0.083)
Overweight & Obese ^†^	1.082	1.110	0.581	0.290	−0.076
	(0.65, 1.79)	(0.77, 1.60)	(0.36, 0.94)	(0.11, 1.79)	(0.072)
*N*	*1881*	*1249*	*1249*	*1249*	*1249*
***WOMEN WITHOUT ANY QUALIFICATION ^c^***					
BMI group					
Underweight ^†^	1.015	0.656 **	0.873	1.359	0.109 ***
	(0.83, 1.24)	(0.47, 0.92)	(0.68, 1.13)	(0.99, 1.87)	(0.039)
Overweight & Obese ^†^	0.741	0.352 **	0.332 ***	0.871	−0.033
	(0.49, 1.13)	(0.18, 0.68)	(0.18, 0.61)	(0.38, 1.98)	(0.071)
*N*	*1745*	*977*	*977*	*977*	*977*

^†^ BMI groups include underweight (BMI < 25), overweight or obese (BMI ≥ 25), normal weight (18.5 < BMI ≤ 25) as references. ^b^ ** *p*-value < 0.05, *** for *p*-value < 0.01. ^c^ The following three qualifications were considered: having any certificates, job training, or internship.

## References

[1] NCD Risk Factor Collaboration (2016). Trends in adult body-mass index in 200 countries from 1975 to 2014: A pooled analysis of 1698 population-based measurement studies with 19·2 million participants. Lancet.

[2] Morgen C.S., Sørensen T.I. (2014). Obesity: Global trends in the prevalence of overweight and obesity. Nat. Rev. Endocrinol..

[3] Lin S.-J. (2016). Examining the relationship between obesity and wages: Empirical evidence from Taiwan. J. Dev. Areas.

[4] Korea Centers for Disease Control and Prevention (1998–2015). National Health and Nutrition Examination Survey.

[5] Rhee S.Y., Park S.W., Kim D.J., Woo J. (2013). Gender disparity in the secular trends for obesity prevalence in Korea: Analyses based on the KNHANES 1998–2009. Korean J. Intern. Med..

[6] Caliendo M., Lee W.-S. (2013). Fat chance! Obesity and the transition from unemployment to employment. Econ. Hum. Biol..

[7] Cavico F.J., Muffler S.C., Mujtaba B.G. (2012). Appearance discrimination in employment. Equal. Divers. Incl..

[8] Wright E.J., Whitehead T.L. (1987). Perceptions of body size and obesity: A selected review of the literature. J. Community Health.

[9] Cachelin F.M., Rebeck R.M., Chung G.H., Pelayo E. (2002). Does ethnicity influence body-size preference? A comparison of body image and body size. Obesity Res..

[10] Ruhm C.J. (2012). Understanding overeating and obesity. J. Health Econ..

[11] Fan M., Jin Y. (2013). Obesity and Self-control: Food Consumption, Physical Activity, and Weight-loss Intention. Appl. Econ. Perspect. Policy.

[12] Strauss R.S. (2000). Childhood obesity and self-esteem. Pediatrics.

[13] Caliendo M., Gehrsitz M. (2016). Obesity and the labor market: A fresh look at the weight penalty. Econ. Hum. Biol..

[14] Chelcea S., Ivan L. (2016). The “Aphrodite effect”: Labor market discrimination based on attractiveness. Psihol. Soc..

[15] Han E., Norton E.C., Stearns S.C. (2009). Weight and wages: Fat versus lean paychecks. Health Econ..

[16] Han E., Kim T.H. (2016). Body Mass Index and Self-employment in South Korea. J. Biosoc. Sci..

[17] Altonji J.G., Pierret C.R. (2001). Employer learning and statistical discrimination. Q. J. Econ..

[18] Kim T.H., Han E. (2017). Height premium for job performance. Econ. Hum. Biol..

[19] Oyer P. (2006). Initial labor market conditions and long-term outcomes for economists. J. Econ. Perspect..

[20] Pinkston J.C. (2017). The dynamic effects of obesity on the wages of young workers. Econ. Hum. Biol..

[21] Kim T.H., Han E. (2015). Impact of body mass on job quality. Econ. Hum. Biol..

[22] Duncan G.J., Holmlund B. (1983). Was Adam Smith Right After All? Another Test of the Theory of Compensating Wage Differentials. J. Labor Econ..

[23] Brown C. (1980). Equalizing differences in the labor market. Q. J. Econ..

[24] Jencks C., Perman L., Rainwater L. (1988). What is a good job? A new measure of labor-market success. Am. J. Sociol..

[25] Centers for Disease Control and Prevention in Korea National Statistics for Health 2012. http://www.index.go.kr/potal/main/EachPage.do?mmenu=2&smenu=4.

[26] World Health Organization (2000). The Asia-Pacific Perspective: Redefining Obesity and Its Treatment.

[27] World Health Organization (2013). Obesity and Overweight Fact Sheet N 311. http://www.who.int/mediacentre/factsheets/fs311/en/em.

[28] Morris S. (2007). The impact of obesity on employment. Labour Econ..

[29] Roehling M.V. (2002). Weight Discrimination in the American Workplace: Ethical Issues and Analysis. J. Bus. Ethics.

[30] Flint S.W., Snook J. (2014). Obesity and discrimination: The next ‘big issue’?. Int. J. Discrimination Law.

[31] Kinge J.M. (2016). Body mass index and employment status: A new look. Econ. Hum. Biol..

[32] Cawley J. (2004). The Impact of Obesity on Wages. J. Hum. Resour..

[33] Lundberg S., Startz R. (1983). Private Discrimination and Social Intervention in Competitive Labor Markets. Am. Econ. Rev..

[34] Spence M. (1973). Job Market Signaling. Q. J. Econ..

[35] Weiss A. (1995). Human capital vs. signalling explanations of wages. J. Econ. Perspect..

[36] Cho D., Choi K. (2015). A Study on the Time-varying Relationships between Korean Economic Growth and Unemployment. J. Ind. Econ. Bus..

[37] Muenster E., Rueger H., Ochsmann E., Letzel S., Toschke A.M. (2011). Association between overweight, obesity and self-perceived job insecurity in German employees. BMC Public Health.

[38] Rowland M.L., Rowland M.L. (1990). Self-reported weight and height. Am. J. Clin. Nutr..

[39] Dhaliwal S.S., Howat P., Bejoy T., Welborn T.A. (2010). Self-reported weight and height for evaluating obesity control programs. Am. J. Health Behav..

[40] Garn S.M., Leonard W.R., Hawthorne V.M. (1986). Three limitations of the body mass index. Am. J. Clin. Nutr..

[41] Burkhauser R.V., Cawley J. (2008). Beyond BMI: The value of more accurate measures of fatness and obesity in social science research. J. Health Econ..

[42] Johansson E., Bockerman P., Kiiskinen U., Heliovaara M. (2009). Obesity and labour market success in Finland: The difference between having a high BMI and being fat. Econ. Hum. Biol..

